# Production of Prosaikogenin F, Prosaikogenin G, Saikogenin F and Saikogenin G by the Recombinant Enzymatic Hydrolysis of Saikosaponin and their Anti-Cancer Effect

**DOI:** 10.3390/molecules27103255

**Published:** 2022-05-19

**Authors:** Ji-Eun Lee, Bong-Kyu Song, Ju-Hyeon Kim, Muhammad-Zubair Siddiqi, Wan-Taek Im

**Affiliations:** 1 Department of Biotechnology, Major in Applied Biotechnology, Hankyong National University, 327 Chungang-no, Anseong-si 17579, Gyeonggi-do, Korea; spaspa0805@naver.com (J.-E.L.); mzsiddiqi1988@gmail.com (M.-Z.S.); 2 AceEMzyme Co., Ltd., Academic Industry Cooperation, 327 Chungang-no, Anseong-si 17579, Gyeonggi-do, Korea; myking0129@naver.com (B.-K.S.); whghldi11@naver.com (J.-H.K.); 3 HK Ginseng Research Center, 327 Chungang-no, Anseong-si 17579, Gyeonggi-do, Korea

**Keywords:** *Bupleurum falcatum*, saikosaponins, prosaikogenin, saikogenin, enzymatic hydrolysis, anticancer

## Abstract

The saponins of *Bupleurum falcatum* L., saikosaponins, are the major components responsible for its pharmacological and biological activities. However, the anti-cancer effects of prosaikogenin and saikogenin, which are glycoside hydrolyzed saikosaponins, are still unknown due to its rarity in plants. In this study, we applied two recombinant glycoside hydrolases that exhibit glycoside cleavage activity with saikosaponins. The two enzymes, BglPm and BglLk, were cloned from *Paenibacillus mucilaginosus* and *Lactobacillus koreensis*, and exhibited good activity between 30–37 °C and pH 6.5–7.0. Saikosaponin A and D were purified and obtained from the crude *B. falcatum* L. extract using preparative high performance liquid chromatography technique. Saikosaponin A and D were converted into saikogenin F via prosaikogenin F, and saikogenin G via prosaikogenin G using enzyme transformation with high β-glycosidase activity. The two saikogenin and two prosaikogenin compounds were purified using a silica column to obtain 78.1, 62.4, 8.3, and 7.5 mg of prosaikogenin F, prosaikogenin G, saikogenin F, and saikogenin G, respectively, each with 98% purity. The anti-cancer effect of the six highly purified saikosaponins was investigated in the human colon cancer cell line HCT 116. The results suggested that saikosaponins and prosaikogenins markedly inhibit the growth of the cancer cell line. Thus, this enzymatic technology could significantly improve the production of saponin metabolites of *B. falcatum* L.

## 1. Introduction

*Bupleurum falcatum* L. is an herbal medicine traditionally used to treat influenza, fever, inflammation, malaria, menstrual disorders, and hepatitis in Asian countries for a long time. Saikosaponins are the major components of *Bupleurum falcatum* L. and are responsible for its biological and pharmacological activities, including anti-inflammatory, anticancer, antidepressant, antiviral, hepatoprotection, immune-regulation and neuromodulation (neuroprotective) effects [1,2,3,4,5,6,7].

**Figure 1:**
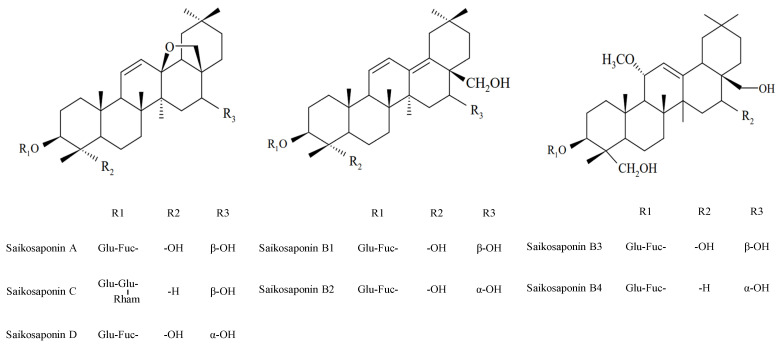
Structure of various saikosaponins.

In recent years, over 100 different triterpenoid saponins have been isolated from *B. falcatum* L.; among them, saikosaponin A, D, C, B1, B2, B3, and B4 (Figure 1) are believed to be responsible for the pharmacological activities of *B. falcatum* L. [8]. Saikosaponins are oleanane-type triterpenoid saponins that are divided into seven types according to their aglycones. Saikosaponin A, D and C are epoxy-ether saikosaponins (type I), whereas saikosaponin B2, with a different aglycone, is heterocyclic diene saikosaponin (type II) [9].

Saikosaponin A and D are important physiologically active ingredients that are well-known for their anti-cancer, anti-inflammatory, and anti-allergic effects [10]. For example, saikosaponins A and D have anti-cancer effects in the SKOV3 and A549 cell lines via a mechanism sensitizing cancer cells to cisplatin-induced cell death [11]. Saikosaponin A also has anti-cancer effects in MDA-MB-231 cells independent of the P53/p21 pathway mechanism and was accompanied by an increased ratio of Bax to Bcl-2 and c-myc levels and activation of caspase-3 [12]. Saikosaponin D decreased the cell proliferation and induced apoptosis both in p53-positive Hep G2 and p53-negative Hep 3B cells [13]. Meanwhile, further study of prosaikogenins and saikogenins is required to better understand the in vivo metabolism. However, it was difficult to obtain prosaikogenins and saikogenins through commercial approach. Many attempts have been made to convert glycoside into its secondary forms, such as by applying physical, chemical, and biotransformation methods to transform ginsenoside.

According to the International Agency for Research on Cancer, colorectal cancer is the third most commonly diagnosed malignancy and the fourth leading cause of cancer death in the world [14]. 5-fluorouracil is the only approved drug for treating advanced colorectal cancer. Although chemotherapy remains the standard treatment, it causes several side effects such as nausea, vomiting, loss of appetite, constipation or diarrhea, and alopecia. Therefore, alternative therapeutic strategies are needed [15].

**Figure 2:**
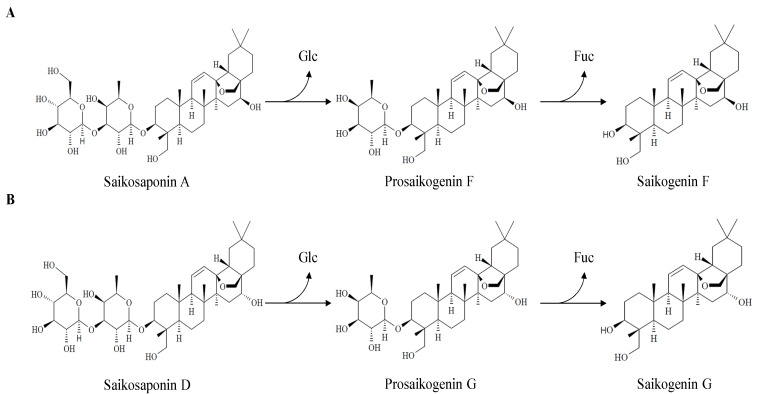
Biotransformation pathway of saikosaponins. (**A**) Biotransformation pathway of saikosaponin A into saikogenin F via prosaikogenin F by BglPm; (**B**) Biotransformation pathway of saikosaponin D into saikogenin G via prosaikogenin G by BglLk.

In this study, we designed an experiment to efficiently produce high purity prosaikogenin and saikogenin using two recombinant enzymes (BglLk and BglPm). The major compounds saikosaponin A (SSA) and D (SSD) were converted into saikogenin F (SGF) via prosaikogenin F (PSF), and saikogenin G (SGG) via prosaikogenin G (PSG) using recombinant β-glucosidase. The recombinant enzyme belongs to the glycoside hydrolase family 1 (GH1) and GH3 and is cloned from *Paenibacillus mucilaginosus* and *Lactobacillus koreensis*, respectively. While only focused on the production of ginsenoside in a previous study, we found that two β-glucosidases are ideal for the production of prosaikogenin F, prosaikogenin G, saikogenin F, and saikogenin G. The recombinant enzymes could hydrolyze the glucose moieties at the C3 positions saikosaponin A and D (Figure 2). Subsequently, pure saikosaponins were used for the in vitro study of anti-cancer effects against colorectal cancer cell HCT 116.

## 2. Results and Discussion

### 2.1. Quantitative Analysis of Bupleurum falcatum *L.*

The results of the HPLC analysis of the saikosaponins were validated as shown in Table 1, where the correlation coefficient (R^2^ > 0.999) indicated good correlations between their peak areas and the concentrations of saikosaponins. As a result of HPLC analysis, the total saikosaponin content of Chinese *Bupleurum falcatum* L. was 6.52~7.10%. It was higher than that of Korean *Bupleurum falcatum* L. (3.32~3.88%). The major saikosaponin was saikosaponin A, saikosaponin C, and saikosaponin D. The quantitative results for the components are shown in Table 2.

### 2.2. Purification of Saikosaponin A and Saikosaponin D with High Purity

To obtain saikosaponin A and D, and additionally fractionate into other components, 7.5 g of saikosaponin extract was purified twice using a silica column. A gradient condition of chloroform:methanol:distilled H_2_O (90:10:10, 80:20:10, 78:32:10, and 65:35:10) was used. A total of 12 g of saikosaponin A and D were obtained from the silica fraction. Saikosaponin A and D, as well as other components, were analyzed through HPLC analysis. (Figure S1B, see Supplementary Materials). The HPLC analysis results of those samples after prep-HPLC showed that fractions 5, 6, and 7 collected from 150 mL under 40% ACN recorded a purity of 98%, and fractions 12 and 13 collected from 150 mL under 55% ACN also recorded a purity of 98%. Subsequently, the same component fractions were combined and concentrated under reduced pressure to obtain 251.2 mg of saikosaponin A and 273.4 mg of saikosaponin D. The chromatographic purity of the saikosaponin A and saikosaponin D was more than 98%, as shown by the HPLC analyses (Figure S2B,C).

### 2.3. Biotransformation of Saikosaponin into Saikogenin via Prosaikogenin

**Figure 3:**
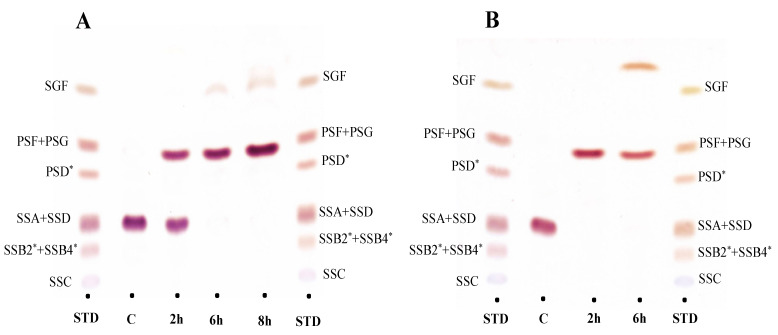
Time-course thin layer chromatography (TLC) analyses of saikosaponin A and D biotransformation by BglPm and BglLk, respectively; (**A**) TLC analysis of biotransformation saikosaponin A by BglPm.; Lane STD, saikosaponin standard; Lane C, substrate saikosaponin A; Lane 2 h, the reaction after two-hours treatment with BglPm; Lane 6 h, the reaction after six-hours treatment with BglPm; Lane 8 h, the reaction after eight-hours treatment with BglPm. (**B**) TLC analysis of biotransformation saikosaponin D by BglLk.; Lane STD, saikosaponin standard; Lane C, substrate saikosaponin D; Lane 2 h, the reaction after two-hours treatment with BglLk; Lane 6 h the reaction after six-hours treatment with BglLk. * “SSB2” means saikosaponin B2; “SSB4” means saikosaponin B4; “PSD” means prosaikogenin D.

**Figure 4:**
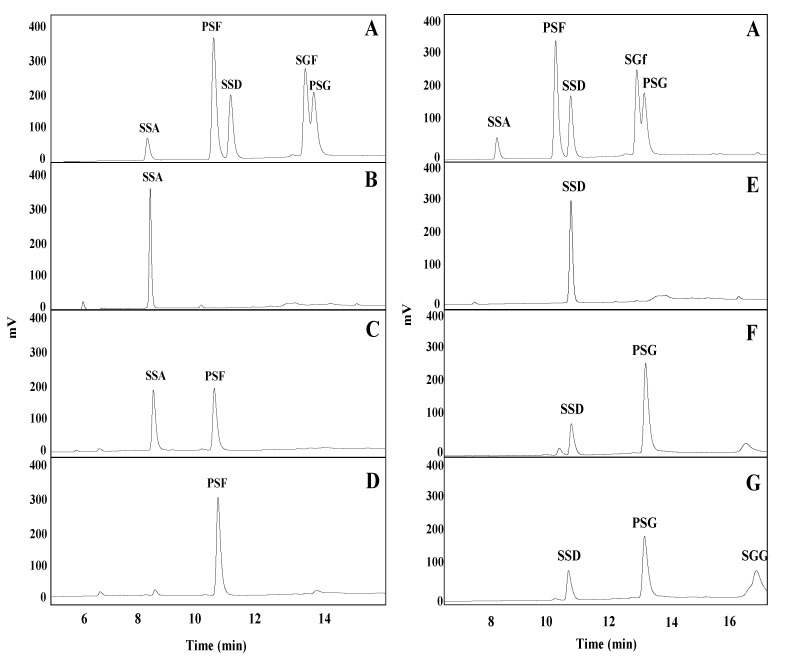
HPLC results of the biotransformation saikosaponin A and saikosaponin D by BglPm and BglLk, respectively (**A**) saikosaponin standard; (**B**) substrate saikosaponin A; (**C**) the reaction after two-hours treatment with BglPm; (**D**) the reaction after eight-hours treatment with BglPm; (**E**) substrate saikosaponin D; (**F**) the reaction after two-hours treatment with BglLk; (**G**) the reaction after six-hours treatment with BglLk.

To observe the biotransformation pathway of saikosaponin A and D using BglPm and BglLk, respectively, TLC and HPLC analyses were performed for a predetermined time period. The peak retention time showed that BglPm completely converted saikosaponin A to prosaikogenin F within 8 h of reaction. After a period of time, a small amount of saikogenin F was formed (Figure 3A and Figure 4D). BglLk converted saikosaponin D to prosaikogenin G within 2 h of reaction, and a small amount of saikogenin G was formed after a longer period of time (Figure 3B and Figure 4F,G).

### 2.4. Purification of Biotransformed Prosaikogenins and Saikogenins

To purify the saikosaponins, 90 mg of prosaikogenin F–saikogenin F mixture was loaded into a silica cartridge. After isocratic chloroform-methanol elution (90:10, *v/v*) 78.1 mg of prosaikogenin F and 8.3 mg of saikogenin F were obtained. Additionally, 72 mg of the prosaikogenin G–saikogenin G mixture was loaded into a silica cartridge. After isocratic chloroform–methanol elution (90:10, *v/v*) 62.4 mg of prosaikogenin G, and 7.5 mg of saikogenin G was obtained. The chromatographic purity of the four saikosaponins was more than 98%, as shown by the HPLC analyses (Figure S2D–F).

Furthermore, we analyzed the six purified saikosaponins by LC/MS. The TIC (total ion chromatogram) of the six purified saikosaponins in negative ion mode is shown in Figure S3. By comparing the retention times, accurate masses, and characteristic LC/MS fragment ions, the six purified saikosaponins were identified. The six purified saikosaponins were identified as saikosaponin A, saikosaponin D, prosaikogenin F, prosaikogenin G, saikogenin F, and saikogenin G.

### 2.5. Anti-Cancer Effect of Saikosaponin, Prosaikogenin, and Saikogenin

**Figure 5:**
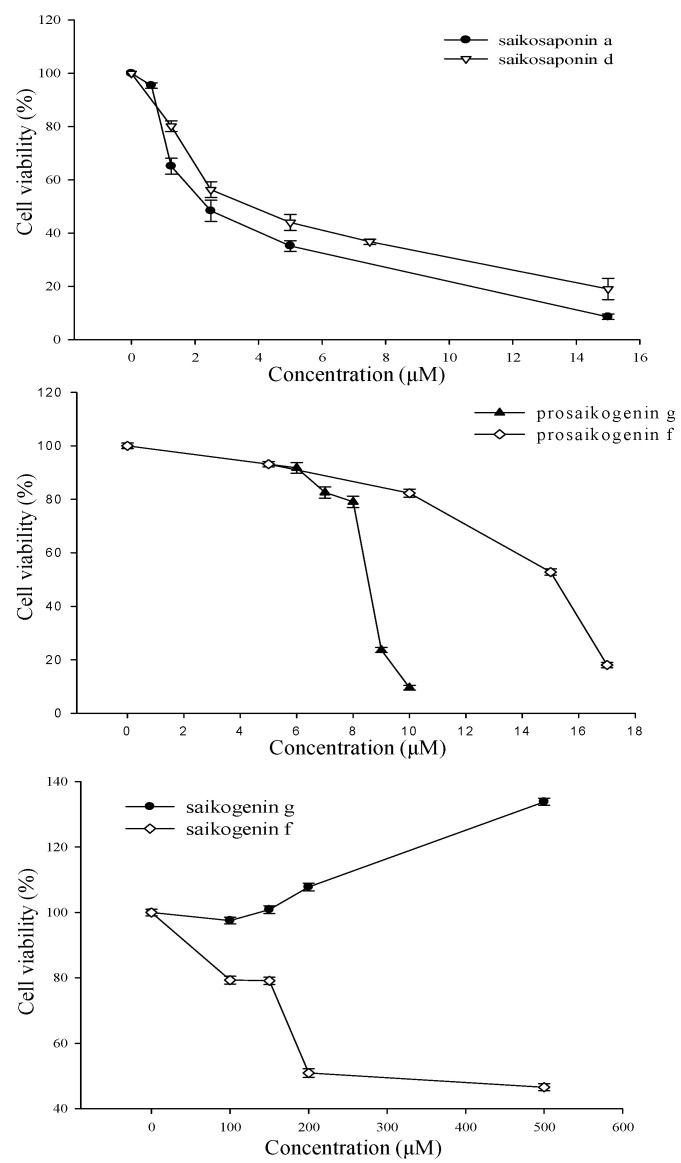
Effect of saikosaponin A, saikosaponin D, prosaikogenin F, prosaikogenin G, saikogenin F, and saikogenin G on proliferation of HCT 116 cells. Cell proliferation was determined by CCK-8 assay.

Treatment with saikosaponin A, saikosaponin D, prosaikogenin F, and prosaikogenin G caused significant cell death in HCT 116 cancer cells at the tested concentrations. However, saikogenin F and G did not show significant anti-cancer effects. The IC_50_ values of saikosaponin A, saikosaponin D, prosaikogenin F, and prosaikogenin G on HCT 116 cancer cells were 2.83, 4.26, 14.21, and 8.49 μM, respectively (Figure 5), which suggests that they exert an anti-cancer effect on HCT 116 cancer cell lines. In contrast, saikogenin G did not inhibit HCT 116 cancer cell line growth at the various concentration ranges within 24 h (Figure 5). Rather, it caused the growth of cancer cells. Saikogenin F only caused the death of cancer cells at a very high concentration (Figure 5); thus, it was considered to not exhibit significant anti-cancer effects.

## 3. Materials and Methods

### 3.1. Materials

Standard of saikosaponin A, saikosaponin C, saikosaponin D, saikosaponin B2, prosaikogenin D, prosaikogenin F, and saikogenin F with 98% purity were purchased from Chengdu Biopurify phytochemicals Ltd. (Chengdu, China). Two recombinant enzymes were used in this enzymatic hydrolysis: BglPm (β-glucosidase from *P. mucilaginosus* KCTC 3870^T^) [16] and BglLk (β-glucosidase from *L. koreensis* KCTC 13530^T^) [17]. Recombinant *Escherichia coli* was grown for protein expression in Luria-Bertani (LB) medium (BD, San Diego, CA, USA) supplemented with ampicillin (100 mg/L) (Gibco, Grand Island, NY, USA). High-performance liquid chromatography (HPLC)-grade reagents and solvents (methanol, acetonitrile, butanol, and chloroform) were obtained from Merck (Darmstadt, Germany). Six kilograms of dried roots of *B. falcatum* L. were extracted twice with 95 L of 50% ethanol. The extract was filtered through filter papers and dried using a rotary evaporator. The resultant dried powder was dissolved in water and loaded onto a glass column, 400 (L) × 115 (D) mm, packed with Diaion HP-20 resin (Mitsubishi Chemical, Tokyo, Japan). Free sugar molecules and unwanted hydrophilic compounds from HP-20 absorbed beads were washed with eight column volumes (CVs) of water, and finally, crude saikosaponins were eluted with four CVs of 95% ethanol. The ethanol extracts were evaporated in vacuo, and the dried crude saikosaponins were used to prepare the saikosaponin mixture.

### 3.2. Quantitative Analysis of Bupleurum falcatum *L.*

Dried *B. falcatum* L. roots were obtained from different countries (Korea and China). Samples were powdered and then extracted with 50 mL of 50% MeOH, which was placed in a shaking incubator for 16 h at 50 °C and 200 rpm. Extracts were then purified using a Sep-pak cartridge (Waters, Worcester County, MA, USA). Six saikosaponins (saikosaponin A, saikosaponin C, saikosaponin D, prosaikogenin F, prosaikogenin G, and saikogenin F) were simultaneously analyzed using the reverse-phase HPLC system at UV 203 nm.

### 3.3. Purification and Separation of Saikosaponin Mixture as Saikosaponin A and Saikosaponin D

The saikosaponin mixture was purified using a Biotage^®^ SNAP KP-Sil 340 g cartridge (Biotage, Uppsala, Sweden). First, the cartridge was flushed with 100% of four CVs of chloroform. Second, the cartridge was flushed with four CVs under the initial gradient condition. Third, the saikosaponin mixture (15 g/200 mL) was then loaded and dissolved under the initial gradient condition. Elution was performed under four gradient conditions with chloroform:methanol:distilled H_2_O (90:10:10, 80:20:10, 78:32:10, 65:35:10; *v/v/v*). Fractions were taken for every 340 mL elution (one-half CV). A saikosaponin A and D mixture (SSAD-Mix) weighing 800 mg was purified using a preparative HPLC system (LC-920, Japan Analytical Instruments, Tokyo, Japan) equipped with a UV/refractive index (RI) detector and a reverse-phase column (octadecylsilane (ODS), 500 × 20 mm; inside diameter (i.d.), 15 m). A gradient solvent system of acetonitrile: double-distilled H_2_O (40:60 and 55:45) was used, and the detection wavelength was set at 203 nm. The SSAD-Mix was dissolved in 100% methanol.

### 3.4. Transformation of the Saikosaponin A and D Using Recombinant Enzymes BglPm and BglLk

Each recombinant enzyme (BglPm and BglLk) gene was inserted into the pGEX-4T-1 GST fusion vector and resulting recombinant plasmid was transformed into E. coli BL21 (DE3), respectively. A 700 mL working volume of LB broth supplemented with 80 mg/mL of ampicillin in a 1 L flask was used to produce recombinant BglPm and BglLk. Flask cultures were maintained at 37 °C in a shaking incubator at 200 rpm. When the OD at 600 nm reached 0.4–0.5, the temperature was reduced to 25 °C (BglPm) and 20 °C (BglLk). After cooling, the recombinant proteins were induced with 0.2 mM (final concentration) IPTG, and the cultures were further incubated for 24 h at the temperatures previously set for their respective proteins. The pH of the cultures was controlled using hydrochloric acid or ammonia solutions. After 24 h, the cells were harvested by centrifugation at 4000 rpm for 20 min (J1050A-T, Hanil Science Medical Co., Ltd., Daejeon, Korea), and the pellets were resuspended in 10 volumes (*w/v*) of 50 mM sodium phosphate buffer (pH 7.0) and disrupted using a digital sonicator (Digital Sonifier S-450D, Branson, Danbury, CT, USA). Two crude recombinant enzymes (BglPm and BglLk) were present in supernatant and used for further experiments.

Prosaikogenin F and saikogenin F were produced in a 500 mL flask reactor with a 200 mL working volume, using half volume of crude BglPm for 8 h at optimum conditions of 37 °C and 50 mM sodium phosphate buffer (pH 7.0) as 1 mg/mL of saikosaponin A. Like previous reactions, prosaikogenin G and saikogenin G were also produced in a 500 mL flask reactor with a 200 mL working volume, using half volume of crude BglLk for 2 h at optimum conditions of 37 °C and 50 mM sodium phosphate buffer (pH 7.0) as 1 mg/mL of saikosaponin D.

### 3.5. Purification of Prosaikogenins and Saikogenins

Each of the biotransformed saikosaponin A and D were purified using a Biotage^®^ SNAP KP-Sil 50 g cartridge (Biotage, Uppsala, Sweden). First, the cartridge was flushed with 100% of four CVs of chloroform. Second, the cartridge was flushed with four CVs under the initial gradient condition. Third, each saikosaponin mixture (90 and 72 mg) was then loaded and dissolved under the initial gradient condition. Elution was performed under isocratic conditions with chloroform:methanol:distilled H_2_O (90:10:10; *v/v/v*). Fractions were taken for every 60 mL elution (one CV).

### 3.6. Cell Culture

The human colorectal cancer cell line HCT 116 was purchased from the Korea Cell Line Bank (KCLB, Seoul, Korea). Cells were maintained in Dulbecco’s Modified Eagle Medium (DMEM, Hyclone, Logan, UT, USA) supplemented with 10% fetal bovine serum (FBS, Hyclone, Logan, UT, USA) and 1% penicillin/streptomycin (Hyclone, Waltham, MA, USA). The HCT-116 cells were cultured in a 5% CO_2_ incubator at 37 °C under a high humidity environment. 

### 3.7. Cell Viability Assay

Cancer cells were first counted, and approximately 5×10^3^ cells per well were seeded in a 96-well cell culture plate (SPL Life Sciences Co., Ltd., Pocheon, Korea). After culturing at 37 °C in a high humidity environment with 5% CO_2_ for 24 h, the culture medium was replaced with a series of concentrations of drugs diluted with the corresponding culture fluid. Three replicates were made for each measurement, and the time of CO_2_ incubation was determined based on the efficiency of each drug. Saikosaponin A (0.625, 1.25, 2.5, 5, 15 µM), saikosaponin D (0.625, 1.25, 2.5, 5, 7.5, and 15 µM), prosaikogenin F (0, 5, 10, 15, and 17 µM), prosaikogenin G (0, 6, 7, 8, 9, and 10 µM), saikogenin F (0, 100, 150, 200, and 500 µM), and saikogenin G (0, 100, 150, and 500 µM) were co-incubated with the cells for 24 h at 37 °C. Finally, 10 µL of the Quanti-MAX^™^ WST-8 Cell Viability Assay Kit (BIOMAX, Seoul, Korea) was added to each well, and the OD at 450 nm was measured using a multifunction microplate reader (SpectraMax Plus384, Molecular Devices, San Jose, CA, USA) after incubation for 1 h at 37 °C. The half-maximal inhibitory concentration (IC_50_) values were calculated by nonlinear regression analysis using the GraphPad Prism 9.3.1 software (San Diego, CA, USA).

### 3.8. Analytic Methods

#### 3.8.1. Thin Layer Chromatography (TLC) Analysis

TLC was performed using silica gel 60 F_254_ plates (Merck, Darmstadt, Germany) with chloroform: methanol: water (70:30:10, lower phase) as the solvent. Spots on TLC plates were identified by spraying with 10% (*v/v*) H_2_SO_4_ followed by visualization by heating at 110 °C for 5 min, followed by comparison with standard saikosaponin.

#### 3.8.2. High-Performance Liquid Chromatography (HPLC) and LC/MS Analysis

Saikosaponins were analyzed using an HPLC system (Younglin Co. Ltd., Seoul, Korea) with a single wavelength UV detector (model 730D), automatic injector, quaternary pump, and the Younglin AutoChro 3000 software for peak identification and integration. The separation was performed on a TeSa-Pak 120 C_18_ column (5 μm, 250 × 4.6 mm i.d.; Taesan Sci, Korea) with a guard column (Eclipse XDB C_18_, 5 μm, 12.5 × 4.6 mm i.d.). The mobile phases were ACN (A) and water (B). Gradient elution was performed as follows: 32% solvent A and 68% solvent B for 8 min; changing to 35% solvent A and 65% solvent B over 8–12 min; changing to 100% solvent A from 12 min; holding at 100% solvent A for 12–18 min; changing to 32% solvent A and 68% solvent B over 18.0–18.1 min; and holding at 32% and 68%, respectively, for 18.1–28 min. The flow rate was 1.0 mL/min. The sample was detected by absorbance at 203 nm (UV 203 nm). LC/MS for purified saikosaponins were analyzed by a Waters/Micromass ZQ single quadrupole mass spectrometer with negative in mode. Ion spray was operated under 6 L N_2_/min, 3.3 Kv, 25 psi, and 300 °C. The spectra were recorded in the m/z range from 400 to 1000.

## 4. Conclusions

In summary, we attempted to extract the saikosaponins from *B. falcatum* L., fractionate the major saikosaponin, and improve the production process of rare saikosaponins through the biotransformation of major saikosaponin A and D. Ninety milligrams of prosaikogenin F and saikogenin F mixture was obtained, whereas 72 mg of prosaikogenin G and saikogenin G mixture was obtained. A total of 78.1 mg of prosaikogenin F with 98.5 ± 0.3% purity (39.1% conversion rate) and 8.3 mg of saikogenin F 98.4 ± 0.3% purity (4.2% conversion rate) was obtained using 90 mg of crude prosaikogenin and saikogenin after strict purification using a silica column. A total of 62.4 mg of prosaikogenin G with 98.7 ± 0.3% purity (31.2% conversion rate) and 7.5 mg of saikogenin G with 98.5 ± 0.3% purity (3.8% conversion rate) were obtained using 72 mg of crude prosaikogenin and saikogenin after strict purification using a silica column. Saikosaponin A, saikosaponin D, prosaikogenin F, and prosaikogenin G showed excellent anti-cancer effects against HCT 116 cancer cell lines.

To the best of our knowledge, this is the first study on the use of recombinant enzyme treatment for the production of rare saikosaponins. This study also demonstrates the potential use of saikosaponins as an anti-cancer supplement in the pharmaceutical industry.

## Figures and Tables

**Table 1:** Calibration curves of saikosaponin standard analysis.

Saikosaponins	Calibration Curves	R^2^
SSC *	y = 0.0835x − 3.1814	0.9999
SSA *	y = 0.1354x + 0.4894	0.9999
PSF *	y = 0.1058x + 0.8263	0.9998
SSD *	y = 0.1589x + 1.3423	0.9999
SGF *	y = 0.0772x − 2.8478	0.9997
PSG *	y = 0.1245x − 0.3113	0.9999

* “SSA” means saikosaponin A; “SSC” means saikosaponin C; “SSD” means saikosaponin D; “PSF” means prosaikogenin F; “PSG” means prosaikogenin G; “SGF” means saikogenin F.

**Table 2:** Contents (mg/g) of six saponins in 2 batches of *Bupleurum falcatum* root (*n* = 3).

Samples	SSC	SSA	PSF	SSD	SGF	PSG
*Bupleurum falcatum* (Korea)	0.47 ± 0.08	2.01 ± 0.08	0.13 ± 0.02	0.76 ± 0.08	0.11 ± 0.01	0.12 ± 0.01
*Bupleurum falcatum* (China)	0.54 ± 0.07	3.77 ± 0.09	0.25 ± 0.05	1.76 ± 0.1	0.11 ± 0.02	0.43 ± 0.01

## Data Availability

Data is contained within the article or Supplementary Materials.

## Supplementary Materials

The following supporting information can be downloaded at: https://www.mdpi.com/article/10.3390/molecules27103255/s1, Figure S1: HPLC analysis of silica purification of *Bupleurum falcatum* L. extract (A) saikosaponin standard; (B) saikosaponin A and D mix after silica prep.; Figure S2: HPLC analysis of purified saikosaponins using prep-HPLC or silica purification (A) saikosaponin standard; (B) saikosaponin A purified using prep-HPLC; (C) saikosaponin D purified using prep-HPLC; (D) prosaikogenin F purified using silica; (E) prosaikogenin G purified using silica; (G) saikogenin F purified using silica; (F) saikogenin G purified using silica; Figure S3: LC/MS spectrum of purified saikosaponins using prep-HPLC or silica purification.

## Sample Availability

Samples of the compounds are not available from the authors.

## References

[1] Xie J.Y., Di H.Y., Li H., Cheng X.Q., Zhang Y.Y., Chen D.F. (2012). Bupleurum chinense DC polysaccharides attenuates lipopolysaccharide-induced acute lung injury in mice. Phytomedicine.

[2] Liu R.Y., Li J.P. (2014). Saikosaponin-d inhibits proliferation of human undifferentiated thyroid carcinoma cells through induction of apoptosis and cell cycle arrest. Eur. Rev. Med. Pharmacol. Sci..

[3] Jin X., Zhang Y., Li Q., Zhao J. (2013). Mechanisms underlying the beneficial effects of Kaiyu Granule for depression. Neural Regen. Res..

[4] Chiang L.C., Ng L.T., Liu L.T., Shieh D.E., Lin C.C. (2003). Cytotoxicity and antihepatitis B virus activities of saikosaponins from Bupleurum species. Planta Med..

[5] Wang C., Zhang T., Cui X., Li S., Zhao X., Zhong X. (2013). Hepatoprotective effects of a Chinese herbal formula, longyin decoction, on carbon-tetrachloride-induced liver injury in chickens. Evid. Based Complement. Altern. Med..

[6] Ying Z.L., Li X.J., Dang H., Wang F., Xu X.Y. (2014). Saikosaponin-d affects the differentiation, maturation and function of monocyte-derived dendritic cells. Exp. Ther. Med..

[7] Zhou X., Cheng H., Xu D., Yin Q., Cheng L., Wang L., Song S., Zhang M. (2014). Attenuation of neuropathic pain by saikosaponin A in a rat model of chronic constriction injury. Neurochem. Res..

[8] Lin T.Y., Chiou C.Y., Chiou S.J. (2013). Putative genes involved in saikosaponin biosynthesis in Bupleurum species. Int. J. Mol. Sci..

[9] Li X., Li X., Huang N., Liu R., Sun R. (2018). A comprehensive review and perspectives on pharmacology and toxicology of saikosaponins. Phytomedicine.

[10] Yuan B., Rui Y., Yongsheng M., Shan Z., Xiaodong Z., Ying L. (2017). A systematic review of the active saikosaponins and extracts isolated from Radix Bupleuri and their applications. Pharm. Biol..

[11] Wang Q., Zheng X., Yang L., Shi F., Gao L., Zhong Y., Sun H., He F., Lin Y., Wang X. (2010). Reactive oxygen species-mediated apoptosis contributes to chemosensitization effect of saikosaponins on cisplatin-induced cytotoxicity in cancer cells. J. Exp. Clin. Cancer Res..

[12] Chen J., Chang N., Chung J., Chen K. (2003). Saikosaponin-A Induces Apoptotic Mechanism in Human Breast MDA-MB-231 and MCF-7 Cancer Cells. Am. J. Chin. Med..

[13] Hsu Y., Kuo P., Chiang L., Lin C. (2004). Involvement of p53, nuclear factor kappaB and Fas/Fas ligand in induction of apoptosis and cell cycle arrest by saikosaponin d in human hepatoma cell lines. Cancer Lett..

[14] Arnold M., Sierra M.S., Laversanne M., Soerjomataram I., Jemal A., Bray F. (2017). Global patterns and trends in colorectal cancer incidence and mortality. Gut.

[15] Gurunathan S., Muhammad Q., Park C., Yoo H., Kim J., Hong K. (2018). Cytotoxic Potential and Molecular Pathway Analysis of Silver Nanoparticles in Human Colon Cancer Cells HCT116. Int. J. Mol. Sci..

[16] Li L., Shin S.Y., Lee S.J., Moon J.S., Im W.T., Han N.S. (2016). Production of Ginsenoside F2 by Using *Lactococcus lactis* with Enhanced Expression of beta-Glucosidase Gene from *Paenibacillus mucilaginosus*. J. Agric. Food Chem..

[17] Kim J.H. (2020). Characterization of Ginsenoside Conversion Ability of BglLk Cloned from Lactobacillus Koreensis and the Enhanced Production of Ginsenoside C-K from PPD Mix Using Four Glycosidases in Series. Master’s Thesis.

