# Supplementary material for: Production of Prosaikogenin F, Prosaikogenin G, Saikogenin F and Saikogenin G by the Recombinant Enzymatic Hydrolysis of Saikosaponin and their Anti-Cancer Effect
Source: Molecules. 2022 May 19;27(10):3255. doi: 10.3390/molecules27103255 (PMC9145717; doi:10.3390/molecules27103255)
Supplement: Supplementary file 1 [file molecules-27-03255-s001.zip › molecules-1713151-supplementary.pdf]

Production of Prosaikogenin F, Prosaikogenin G, Saikogenin F and Saikogenin G by the recombinant enzymatic hydrolysis of Saikosaponin And their anti-cancer effect

Ji Eun Lee[1] and Wan-Taek Im[1,2,3]*

[1]Major in Applied Biotechnology, Hankyong National University, 327 Chungang-no Anseong-si, Gyeonggi-do 17579, Republic of Korea

[2]AceEMzyme Co., Ltd., Academic Industry Cooperation, 327 Chungang-no Anseong-si, Gyeonggi-do 17579, Republic of Korea

[3]HK Ginseng Research Center, 327 Chungang-no Anseong-si, Gyeonggi-do 17579, Republic of Korea

*Corresponding author

E-mail: wandra@hknu.ac.kr

Phone: +82 31 6705335; Fax: +82 31 6705339;

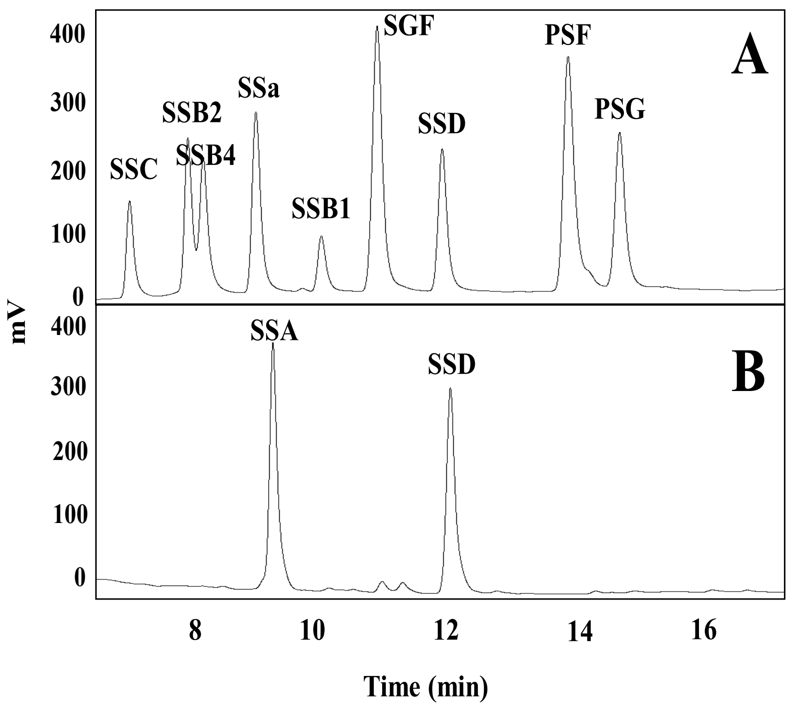

Supplementary Figure S1. HPLC analysis of silica purification of *Bupleurum falcatum* L. extract **(A)** saikosaponin standard; **(B)** saikosaponin A and D mix after silica prep.

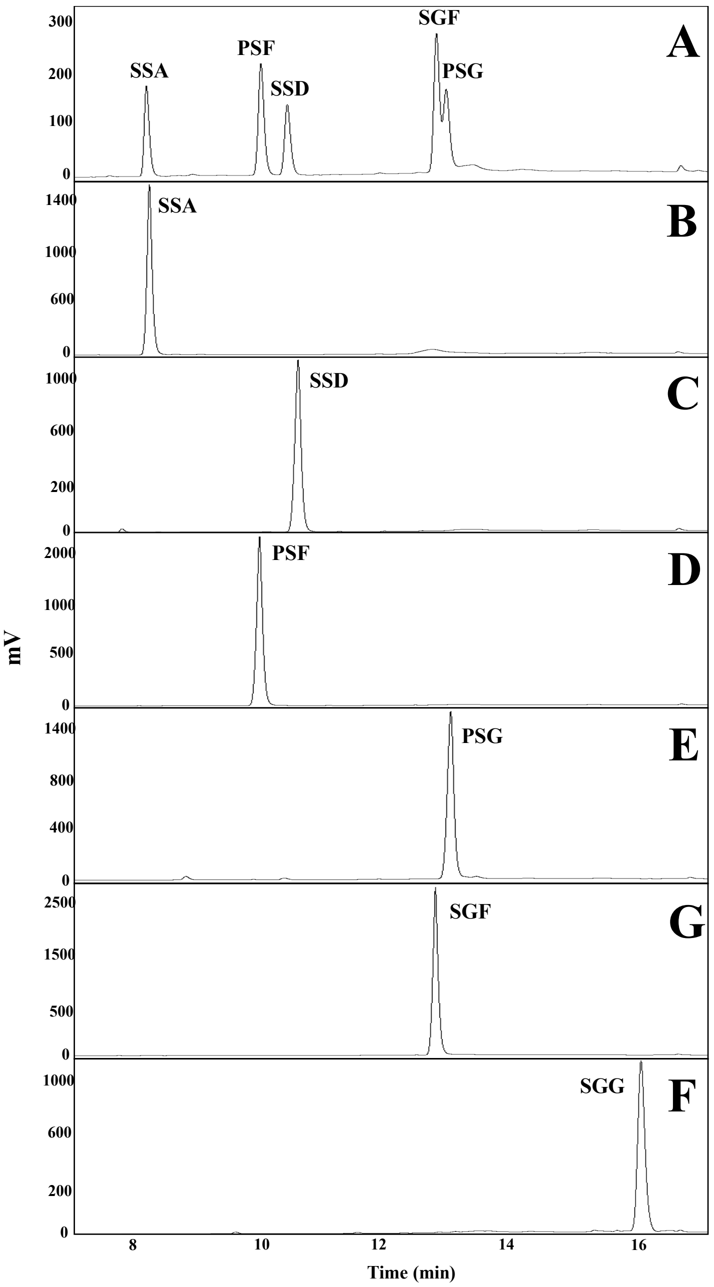

Supplementary Figure S2. HPLC analysis of purified saikosaponins using prep-HPLC or silica purification (A) saikosaponin standard; (B) saikosaponin A purified using prep-HPLC; (C) saikosaponin D purified using prep-HPLC; (D) prosaikogenin F purified using silica; (E) prosaikogenin G purified using silica; (G) saikogenin F purified using silica; (F) saikogenin G purified using silica.

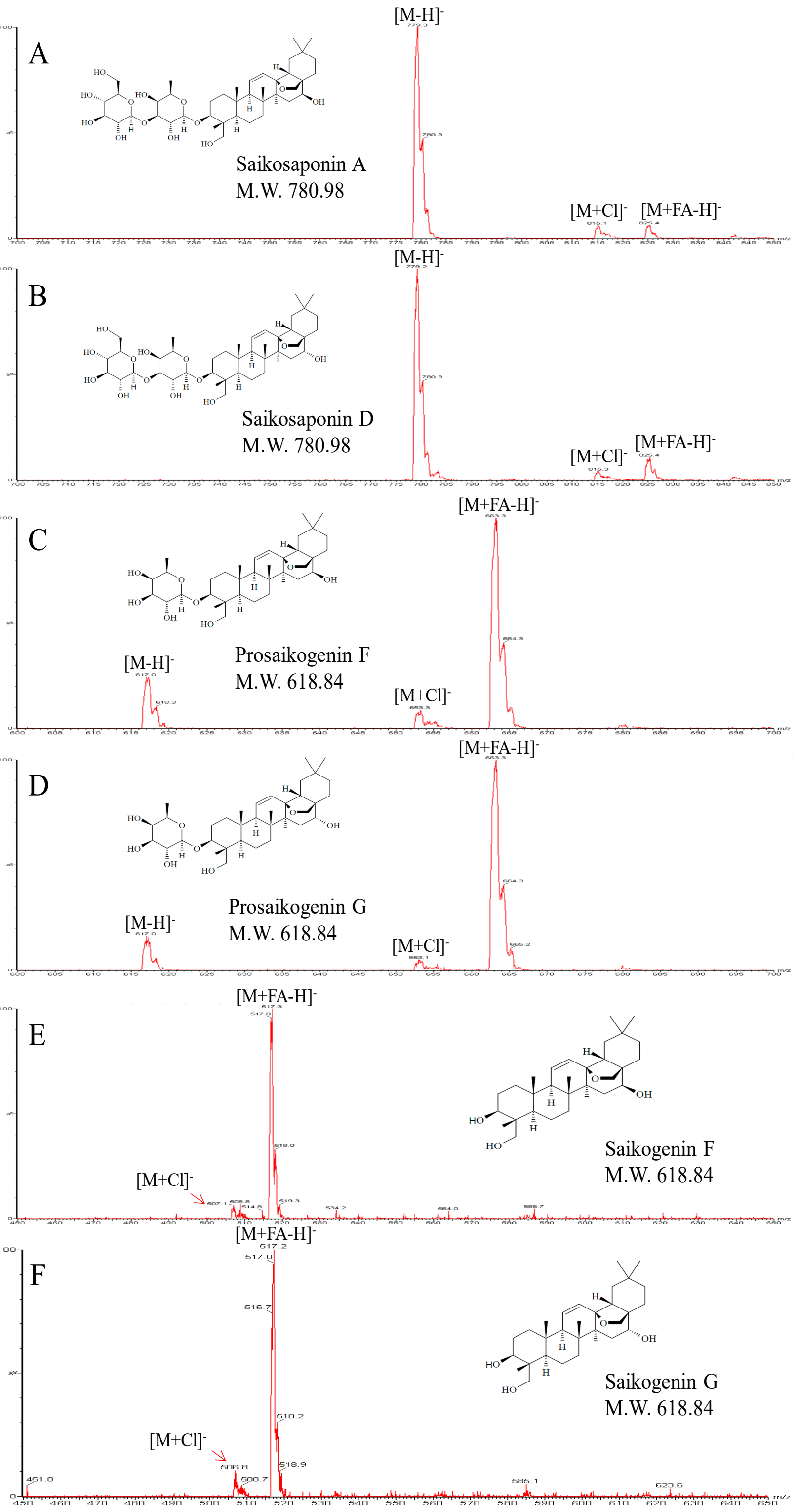

Supplementary Figure S3. LC/MS spectrum of purified saikosaponins using prep-HPLC or silica purification.
